# Grazing effect on grasslands escalated by abnormal precipitations in Inner Mongolia

**DOI:** 10.1002/ece3.4331

**Published:** 2018-07-22

**Authors:** Maowei Liang, Jiquan Chen, Elise S. Gornish, Xue Bai, Zhiyong Li, Cunzhu Liang

**Affiliations:** ^1^ School of Ecology and Environment Inner Mongolia University Hohhot China; ^2^ Department of Geography, Environment, and Spatial Sciences Center for Global Change and Earth Observations Michigan State University East Lansing Michigan; ^3^ School of Natural Resources and the Environment The University of Arizona Tucson Arizona; ^4^ School of Forest Resources University of Maine Orono Maine

**Keywords:** drought, ecosystem function, Mongolian Plateau, plant competition and facilitation, plant functional group, plant–climate interactions, plant–herbivore interaction, temperate grasslands

## Abstract

Grazing effects on arid and semi‐arid grasslands can be constrained by aridity. Plant functional groups (PFGs) are the most basic component of community structure (CS) and biodiversity & ecosystem function (BEF). They have been suggested as identity‐dependent in quantifying the response to grazing intensity and drought severity. Here, we examine how the relationships among PFGs, CS, BEF, and grazing intensity are driven by climatic drought. We conducted a manipulative experiment with three grazing intensities in 2012 (nondrought year) and 2013 (drought year). We classified 62 herbaceous plants into four functional groups based on their life forms. We used the relative species abundance of PFGs to quantify the effects of grazing and drought, and to explore the mechanisms for the pathway correlations using structural equation models (SEM) among PFGs, CS, and BEF directly or indirectly. Grazers consistently favored the perennial forbs (e.g., palatable or nutritious plants), decreasing the plants’ relative abundance by 23%–38%. Drought decreased the relative abundance of ephemeral plants by 42 ± 13%; and increased perennial forbs by 20 ± 7% and graminoids by 80 ± 31%. SEM confirmed that annuals and biennials had negative correlations with the other three PFGs, with perennial bunchgrasses facilitated by perennial rhizome grass. Moreover, the contributions of grazing to community structure (i.e., canopy height) were 1.6–6.1 times those from drought, whereas drought effect on community species richness was 3.6 times of the grazing treatment. Lastly, the interactive effects of grazing and drought on BEF were greater than either alone; particularly, drought escalated grazing damage on primary production. *Synthesis*. The responses of PFGs, CS, and BEF to grazing and drought were identity‐dependent, suggesting that grazing and drought regulation of plant functional groups might be a way to shape ecosystem structure and function in grasslands.

## 1 INTRODUCTION

Grazing and climate change are the two most critical driving forces shaping the ecosystem structure and function of grasslands (Koerner & Collins, 2014; McNaughton, 1985; Milchunas & Lauenroth, 1993). Currently, global environmental change is broadly recognized as a key element in plant‐herbivore interactions (Asner, Elmore, Olander, Martin, & Harris, 2004; Borer, Grace, Harpole, MacDougall, & Seabloom, 2017; Stein, Harpole, & Suding, 2016). In particular, drought in water‐limited ecosystems appears to exacerbate the effects of grazing (Dangal et al., 2016; Eldridge, Poore, Ruiz‐Colmenero, Letnic, & Soliveres, 2016). For example, increased grazing intensities can lead to a substantial decline in plant diversity, primary production (Biondini, Patton, & Nyren, 1998; Fetzel, Havlik, Herrero, & Erb, 2017), and resistance (Shao et al., 2017). These negative consequences have also been found for the grasslands extensively (Olff & Ritchie, 1998). However, conflicting results have been reported that drought may not be a primary driver in comparison with grazing in shaping grassland plant community structure and ecosystem processes (Koerner & Collins, 2014). Consequently, a grazing optimization hypothesis was proposed as “moderate grazing promotes primary production” (McNaughton, 1979), with natural herbivore grazing leading to higher biodiversity (Collins, Knapp, Briggs, Blair, & Steinauer, 1998; Noy‐Meir, 1995). Our literature search suggests that these seemingly contradictory findings need to be further examined through sound experiments and quantitative analyses on the complex driving mechanisms of climatic and grazing effects in grasslands.

Evidence indicates that plant response to grazing and environmental change is species‐specific, or functionally identity‐dependent (Augustine & McNaughton, 1998; Bai, Han, Wu, Chen, & Li, 2004; Lavorel, McIntyre, Landsberg, & Forbes, 1997; Milchunas et al., 1993; Tilman et al., 1997). For example, large herbivores prefer perennial plants to annuals or biennials, tall plants to short plants, and erect plants to prostrate plants (Díaz et al., 2007; McIntyre & Lavorel, 2001; Milchunas, Sala, & Lauenroth, 1988; Noy‐Meir, Gutman, & Kaplan, 1989). Additionally, grazing can either cause an increase or a decrease in the proportion of dominant species or functional groups (e.g., grasses vs. forbs), depending on the environmental conditions (Bai et al., 2012; Biondini et al., 1998; Collins et al., 1998; Gornish & Ambroso dos Santos, 2016). Meanwhile, plants or plant functional groups can have facilitative interactions or compensatory effects to reduce external pressures on ecosystem structure and functioning (Bai et al., 2004; Maestre, Callaway, Valladares, & Lortie, 2009; Pan et al., 2016). This is because different plant species or functional groups have unique tolerances and avoidance strategies in the face of environmental stress (Li et al., 2015; Ma et al., 2010; Yan et al., 2015), particularly in grazed grasslands (Augustine & McNaughton, 1998; Bai et al., 2012; Pérez‐Camacho et al., 2012; Su et al., 2017; Zheng, Li, Lan, Ren, & Wang, 2015). Despite these bodies of work, it remains unclear how the relationships among plant functional groups (PFGs), plant diversity, primary productivity, and grazing intensity are interactively driven by drought in semi‐arid grasslands.

To fill these knowledge gaps, we designed a field grazing experiment to quantify how grazing and drought collectively affect PFGs, community structure (CS), and biodiversity & ecosystem function (BEF), including their effects on the relationships among PFGs, CS, and BEF. We conducted this study in a typical steppe grassland on the Mongolian Plateau and examined the changes in PFGs, species richness, density, height, and residual live standing aboveground biomass monthly in the growing season (July–September) in a wet (2012) and a dry (2013) year, with the dry year was treated as a drought treatment. We hypothesized that: (a) the effects of grazing on PFGs, CS, and BEF would be dependent of the hydrological condition (i.e., drought), and (b) the interactive effects of grazing and drought may further change the relationships among PFGs, CS and BEF because of within‐community processes (e.g., facilitations).

## 2 MATERIALS AND METHODS

### 2.1 Study site

The study was conducted in a typical steppe grassland located in Eastern Xilinhot, Inner Mongolia, China (44°08′N, 116°19′E, 1,129 m). Between 1953 and 2013, the mean annual air temperature was 2.4°C and mean annual precipitation was 280 mm, with 80%–90% of the precipitation occurring during the growing season (May–September). For our study period, the annual total precipitation was 517.7 mm in 2012 (i.e., a relatively wet year), with 434.9 mm falling during the growing season; the annual precipitation in 2013 was 273.4 mm (i.e., a relatively dry year), with 234.7 mm during the growing season (Liang et al., 2016). The dominant plant species included *Stipa grandis* P. Smirn and *Leymus chinensis* (Trin.) Tzvel. *Cleistogenes squarrosa* (Trin.) Keng, *Anemarrhena asphodeloides* Bunge and *Agropyron cristatum* (L.) Gaertn. were also present, with a total of 65 species recorded in the study area (Supporting Information Table S1).

### 2.2 Grazing experiment and sampling

The study site has experienced continuously nomadic‐grazing with a stocking rate of ~0.55 sheep/ha since 2000. We installed an experiment with two levels of grazing intensity: moderate grazing with 6.0 ± 0.5 hr per day (0.55 sheep/ha), and light grazing with 2.0 ± 0.5 hr/day (0.55 sheep/ha) based on information gained from an interview with the herdsman in charge of livestock management. We considered that the habitat already has been degraded because of the grazing lagged effects; therefore, we choose a control plot (no‐grazing pasture) that had been fenced for passive restoration for 5 years located 2 km from the grazing plots (Liang et al., 2016). The data were collected in 3 months (July–September) during the growing season in the nondrought year (2012) and the drought (2013) year; and 10 plots (1 m × 1 m quadrat) in each treatment pasture transect were randomly placed and sampled, providing us with 30 replicates per grazing treatment.

### 2.3 Community structure

After grazing performance, species richness in each quadrat (1 m × 1 m) was tallied as the total number of plant species (no. species/m^2^); the plant stand density was calculated by dividing the total number of individual plants by quadrat size (no. plants/m^2^). Plant canopy height (cm) was the mean value of all of the plant species that were measured, including the height of reproductive shoot and vegetative shoot.

### 2.4 Biodiversity and ecosystem functions

We calculated the Shannon–Weiner Index using plant stand density in the “vegan” R package (Oksanen et al., 2013). The Rao's Q was used for functional diversity using the “FD” R package (Laliberté, Legendre, Shipley, & Laliberté, 2014), because it includes not only species richness and density but also multiple traits (Botta‐Dukát, 2005; Laliberté & Legendre, 2010), including plant functional types, plant canopy height, wet matter, and dry matter. All plants were harvested independently at ground level in each quadrat, weighed, and then dried in an oven for 48 hr (65°C) to measure dry matter of residual live standing biomass. Accumulated value of dry matter in all plants was used for the aboveground biomass (g/m^2^; Su et al., 2017).

### 2.5 Plant functional groups

The 65 recorded plant species were classified into five PFGs: perennial bunchgrasses, perennial rhizome grass, perennial forbs, annuals and biennials, and shrubs and semi‐shrubs (Supporting Information Table S1). We ultimately did not use the shrub group for statistical analysis because of its low coverage and frequency.

### 2.6 Statistical analysis

As it virtually impossible to replicate the treatments (i.e., the large grazing experiment) at a landscape level, we treated the monthly observations as the replication (*n* = 3), an approach that has been used in several previous studies (Bai et al., 2004; Li et al., 2015; Ma et al., 2010; Yan et al., 2015). Additionally, the drought treatment was referred from Milchunas, Lauenroth, Chapman, and Kazempour (1989). Here, we considered the relative species abundance of PFGs in the community—the ratio of the total number of each PFGs’ species to the total number of species in each quadrat (Garnier et al., 2004)—as a dependent variable for PFGs in our the statistical analyses.

We performed two‐way repeated measures ANOVA to test the fixed effects of grazing, drought, and their interactions on PFGs (perennial bunchgrasses, perennial rhizome grass, perennial forbs, annuals and biennials), CS (species richness, plant stand density, and plant canopy height), and BEF (species diversity, functional diversity, and aboveground biomass), with monthly measurement as a random effect. Secondly, we conducted multiple comparisons using the Tukey's multiple—range test to quantify the effects of drought on each dependent variable by grazing level.

To address our second question, we employed structural equation modeling (SEM) to explore the mechanisms of how grazing and drought either directly affected the path coefficients in BEF and CS, respectively, or indirectly by changing PFGs using AMOS 21 software (IBM, Chicago, IL, USA). Model fitting was based on *χ* ^2^, Comparative Fit Index, Root Mean Square Error of Approximation, and Akaike Information Criteria (Fan et al., 2016; Grace, 2006). All analyses were programmed in R v 3.4.0 (R Development Core Team 2017) unless otherwise noted.

## 3 RESULTS

### 3.1 Coupled effects of grazing and drought

**Figure 1:**
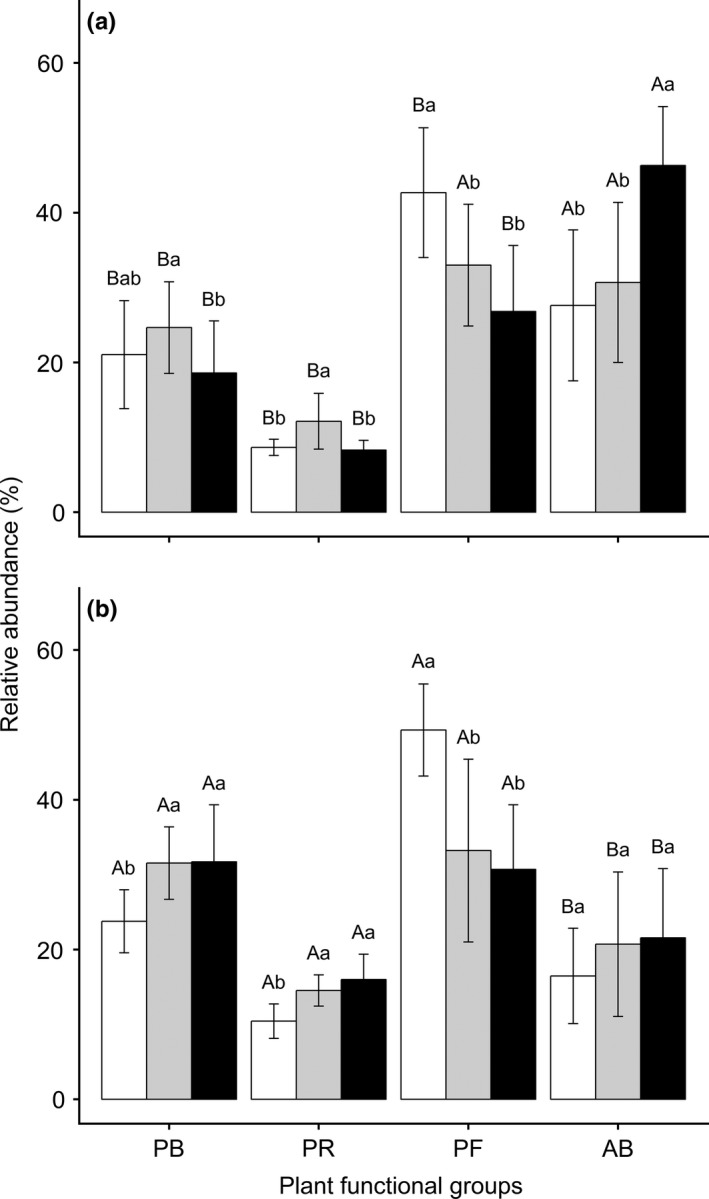
Results of Tukey's test of the relative abundance (Mean ± *SD*) of PFGs in different experimental treatments: (a) in 2012 (nondrought year) and (b) in 2013 (drought year); the white, gray, and black bars represent no‐grazing, light‐grazing, and moderate‐grazing treatment, respectively. PB is perennial bunchgrasses, PR is perennial rhizome grass, PF is perennial forbs, AB is annuals and biennials. Lowercase letters represent significant differences among the grazing treatments in the same drought treatment based on the Tukey's‐range test, and capital letters indicate a significant difference between the nondrought and drought treatments in same grazing intensity from One‐way ANOVA (*p *<* *0.05)

Grazing, drought, and their interactions significantly (*p *<* *0.05) affected the relative abundance of all four PFGs, except for the interactive effect on perennial forbs (*p *=* *0.088; Table 1). The contribution from grazing was 7.3 times of that from drought on perennial forbs. Alternatively, the effects of drought on perennial grasses (bunchgrasses and rhizome grass) were 3.7–5.6 times that of grazing. Surprisingly, the interactive effects of grazing and drought on all PFGs were relatively low (2.7%–9.0%). In 2012 (nondrought year), the relative abundance of perennial forbs decreased by 23%–37% with grazing intensity in contrast to a 11%–68% increase in annuals and biennials. For the perennial bunchgrasses and perennial rhizome grass, the light‐grazing treatment produced the highest relative abundances (*p *<* *0.05; Figure 1a). However, the perennial forbs had a similarly decreasing (33%–38%) in 2013 (drought year) with 2012 in response to the grazing, while the other three PFGs showed trends of increasing abundances with grazing intensity (Figure 1b).

**Table 1:** Results (*F‐*value*s* and contribution percent (i.e., SS_*i*_/SST)) of two‐way ANOVA on the effects of grazing (*df* = 2), drought (year, *df* = 1) and their interactive effects (*df* = 2) on relative abundance of plant functional groups (i.e., perennial bunchgrasses, perennial rhizome grass, perennial forbs, annuals and biennials), community structures (i.e., species richness, stand density, canopy height), and biodiversity and ecosystem functions (i.e., species diversity, functional diversity, and aboveground biomass), with a random effect of month. In the context of ANOVA, SS_G_ is the sum of square by grazing, SS_D_ is the sum of square by drought, and SS_G×D_ is the sum of square by interaction, SST is total the sum of squares. Significance at 95% confidence level: *p <* *0.05; **p <* *0.01; ***p <* *0.001

	Grazing		Drought		Grazing × Drought	
	*F*	SS_G_/SST	*F*	SS_D_/SST	*F*	SS_G×D_/SST
Relative abundance of PFGs						
Perennial bunchgrasses (%)	18.38	10.52***	135.21	38.70***	10.22	5.85***
Perennial rhizome grass (%)	13.27	7.82***	149.11	43.94***	3.34	1.97*
Perennial forbs (%)	34.57	28.74***	9.45	3.93**	**2.47**	**2.06**
Annuals/biennials (%)	29.39	10.69***	326.31	59.32***	3.97	1.44*
Community structure						
Species richness (species/m^2^)	36.28	14.11***	259.72	50.48***	12.61	4.90***
Stand density (plants/m^2^)	44.86	29.95***	14.63	4.89***	19.11	12.75***
Canopy height (cm)	26.60	21.49***	32.97	13.32***	**2.16**	**1.75**
Biodiversity and ecosystem functions						
Species diversity (*H*′)	7.49	5.26***	49.01	17.22***	31.82	22.36***
Functional diversity (Rao's Q)	**2.33**	**2.21**	9.59	4.54***	20.06	18.98***
Aboveground biomass (g/m^2^)	67.60	27.94***	66.07	13.65***	62.81	25.96***

The bold values denote insignificant (*P* > 0.05) effects of grazing, drought, and interactions.

**Figure 2:**
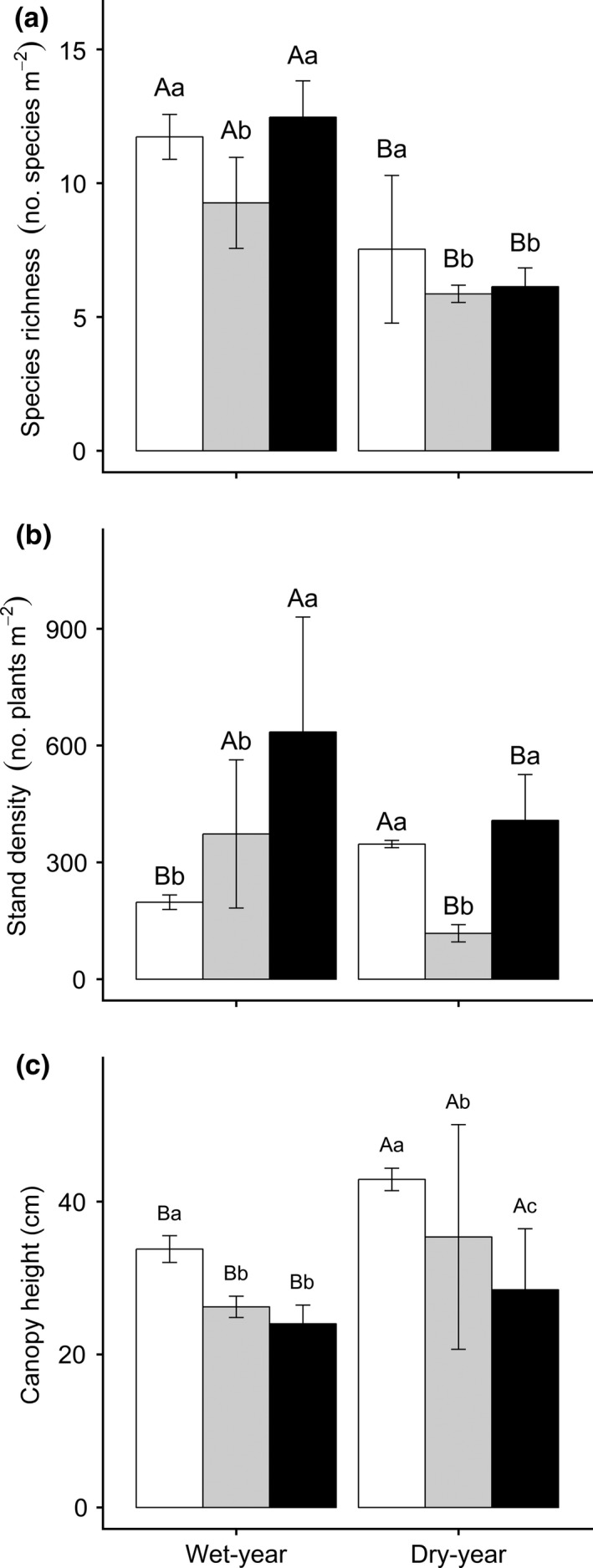
Results of Tukey's test of plant species richness (a), stand density (b), and canopy height (c) in different experimental treatments (Mean ± *SD*). Shown are a Tukey's‐range test among the different grazing treatments in the same drought treatment (*p *<* *0.05) and a One‐way ANOVA between 2012 (nondrought year) and 2013 (drought year) within the same grazing treatment (*p *<* *0.05). The white, gray, and black bars are for no‐grazing, light‐grazing, and moderate‐grazing treatment, respectively

Grazing and drought also significantly altered community structure (*p *<* *0.05), while without significant interactive effects on canopy height (*p *=* *0.119; Table 1). The drought effect on species richness was 3.6 times that of the grazing treatment, whereas the contributions from grazing to plant stand density and canopy height were higher than those from drought (1.6–6.1 times). Similarly, the interactive effects were the lowest for species richness and canopy height (1.8%–4.9%), but the interactive effect on stand density was 2.6 times that of the drought effect and approximately half of the grazing effect (Table 1), suggesting that the differential underlying regulations in the plant community composition (species richness) and structure (plant stand density and canopy height) existed in response to grazing and drought. Specifically, a 21% decrease in plant species richness compared to the no‐grazing treatment in 2012 was caused by light‐grazing (*p *<* *0.001); a 220% increase in plant stand density and a 29% decrease in plant canopy height were caused by moderate‐grazing (*p *<* *0.001). In 2013, a 22% reduction in species richness and a 34% decrease in canopy height was observed in the moderate‐grazing treatment (*p *<* *0.001), and a 66% decrease in plant stand density were found in the light‐grazing treatment (*p *<* *0.001). In sum, grazing consistently reduced canopy height, although canopy heights in all plots were unexpectedly 19%–35% higher in the dry year than in the wet year (*p *<* *0.05; Figure 2).

**Figure 3:**
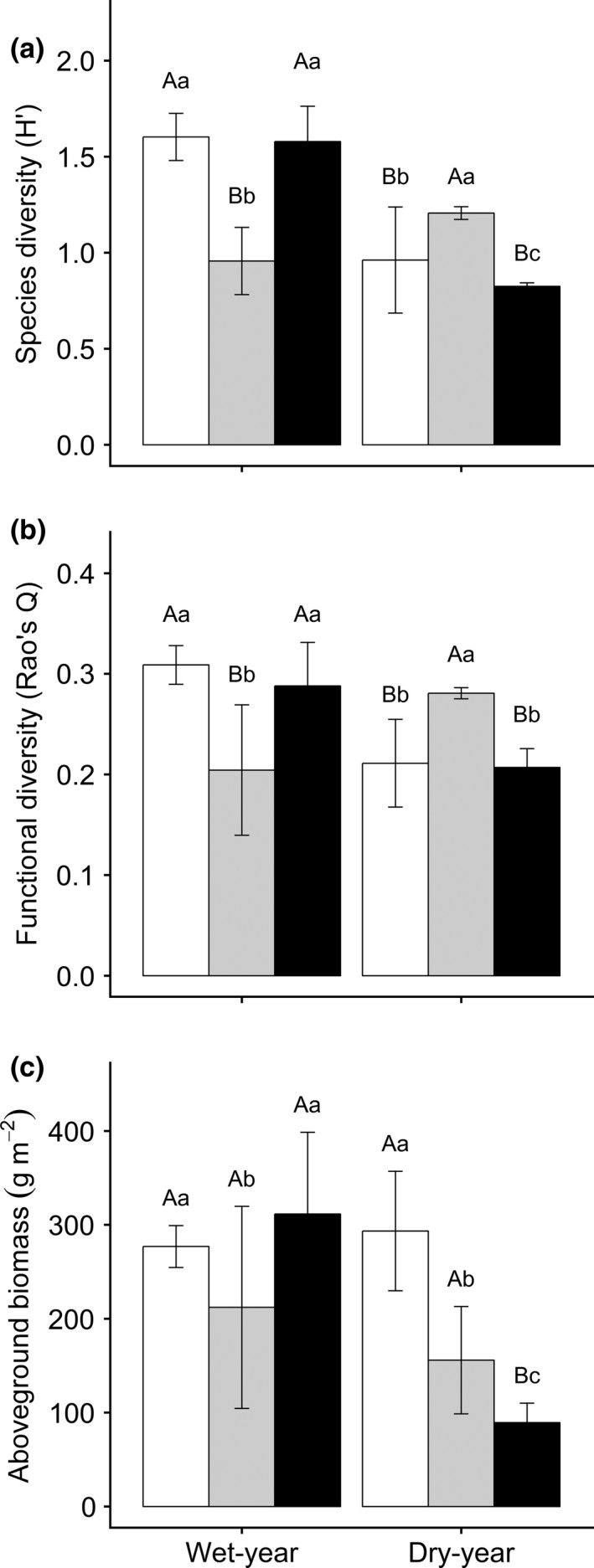
Results of Tukey's test of plant species diversity (a), functional diversity (b), and aboveground biomass (c) in different experimental treatments (Mean ± *SD*). Shown are a Tukey's‐range test among the grazing treatments in the same drought treatment (*p *<* *0.05) and a One‐way ANOVA between 2012 (nondrought year) and 2013 (drought year) within the same grazing treatment (*p *<* *0.05). The white, gray, and black bars are for no‐grazing, light‐grazing, and moderate‐grazing treatment, respectively

Grazing and drought interactively affected biodiversity & ecosystem functions (*p *<* *0.001); however, there were no‐grazing effects on functional diversity (*p *=* *0.100; Table 1). The contribution of drought to species and functional diversity was 2.1 and 3.3 times that from grazing. However, the grazing effect on aboveground biomass was 2.1 times that of drought. Additionally, the interactive effects on BEF were relatively high (19%–26%) in comparison with grazing and drought effects, respectively. In the wet year, the light‐grazing treatment reduced (*p *<* *0.001) 40% species diversity, 34% functional diversity, and 22% aboveground biomass, whereas no changes of these variables occurred in the moderate‐grazing treatment compared to no grazing. In the dry year, light‐grazing resulted in a 25% increase in species diversity and a 33% increase in functional diversity despite severe damage of aboveground biomass (47%). Moderate grazing caused a decrease (*p *<* *0.001) in species diversity by 14% and aboveground biomass by 70% (Figure 3).

### 3.2 Predicting CS and BEF by regulated PFGs in grazing and drought

**Figure 4:**
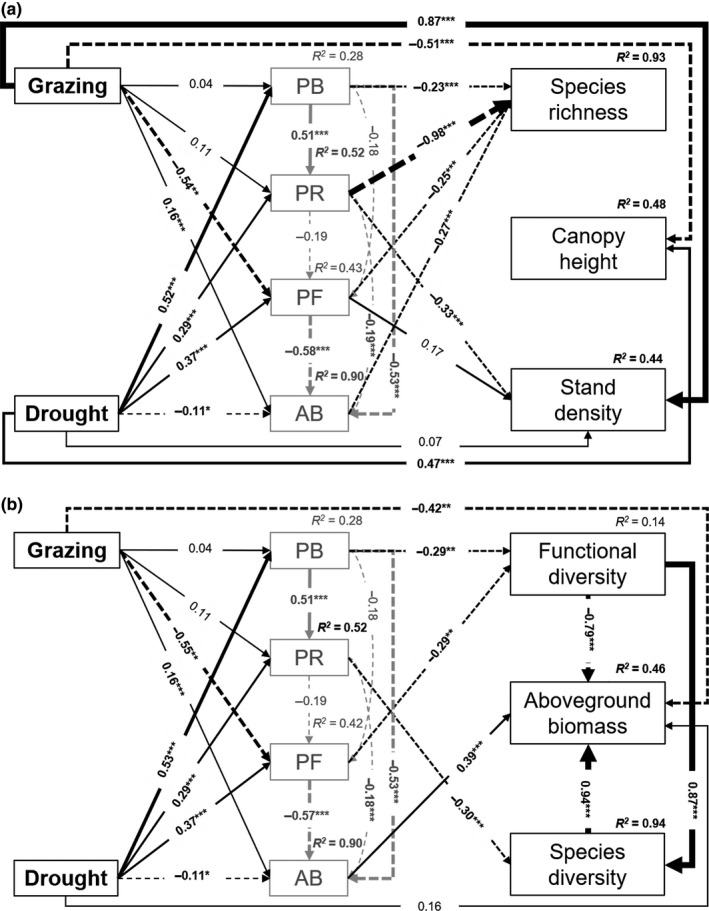
Structural equation modeling (SEM) of grazing and drought to predict CS and BEF by regulating PFGs (*n* = 108). Shown are (a) CS (Chi‐square (*χ* ^2^) = 17.910, degrees of freedom = 12, probability level = 0.118, Comparative Fit Index = 0.992, Root Mean Square Error of Approximation = 0.068, Akaike Information Criteria = 83.910), which include species richness, stand density, and canopy height; (b) BEF (Chi‐square (*χ* ^2^) = 14.809, degrees of freedom = 13, probability level = 0.319, Comparative Fit Index = 0.998, Root Mean Square Error of Approximation = 0.036, Akaike Information Criteria = 78.809), which was referred as species diversity, functional diversity, and aboveground biomass. The PFGs include perennial bunchgrasses—PB, perennial rhizome grass—PR, perennial forbs—PF, and annuals and biennials—AB. Solid and dashed arrows represent positive and negative pathways, respectively. Numbers indicate the standardized path correlation coefficients (*r*). Gray arrows and numbers only demonstrate correlations among PFGs. Significant at 95% confidence level: *p <* *0.05; **p <* *0.01; ***p <* *0.001

The SEM revealed the complex regulations of grazing and drought on the PFGs (Figure 4). In particular, the correlated pathways from grazing to perennial forbs (*r *=* *−0.54) and annuals and biennials (*r *=* *0.16) were apparent (*p *<* *0.001). However, the correlated pathways from drought to all four PFGs (perennial bunchgrasses: *r *=* *0.52, *p *<* *0.001; perennial rhizome grass: *r *=* *0.29, *p *<* *0.001; perennial forbs: *r *=* *0.37, *p *<* *0.001; annuals and biennials: *r *=* *−0.10, *p *=* *0.010) were significant. Perennial bunchgrasses were positively correlated with perennial rhizome grass (*r *=* *0.51, *p *<* *0.001), but the correlated pathways from perennial bunchgrasses (*r *=* *0.18, *p *=* *0.074) and perennial rhizome grass (*r *=* *−0.17, *p *=* *0.075) to perennial forbs were not significant. Annuals and biennials had significantly (*p *<* *0.001) negative correlated pathways from perennial bunchgrasses (*r *=* *−0.53), perennial rhizome grass (*r *=* *−0.18), and perennial forbs (*r *=* *−0.57).

Our SEM also indicted that the PFGs explained 93% of the variation in plant species richness, with negative correlated pathways with all four PFGs directly (*p *<* *0.001). Only 44% of the variation in plant stand density was explained (perennial rhizome grass: *r *=* *−0.33, *p *<* *0.001; perennial forbs: *r *=* *0.17, *p *=* *0.064) despite the joint effect of grazing (*r *=* *0.87, *p *<* *0.001) and drought (*r *=* *0.07, *p *=* *0.476); but grazing (*r *=* *0.07, *p *=* *0.476) and drought explained 48% of the variation in plant canopy height without PFGs’ pathways (Figure 4a). Additionally, the SEM revealed that the PFGs contributed 14% of the variation in functional diversity (perennial bunchgrasses: *r *=* *−0.29, *p *=* *0.002; perennial forbs: *r *=* *−0.29, *p *=* *0.008); 94% of the variation in species diversity was explained by perennial rhizome grass (*r *=* *−0.30, *p *<* *0.001) and functional diversity (*r *=* *0.87, *p *<* *0.001), and 46% of the variation in aboveground biomass was attributed to annuals and biennials (*r *=* *0.39, *p *<* *0.001), species diversity (*r *=* *0.94, *p *<* *0.001), functional diversity (*r *=* *−0.79, *p *<* *0.001), grazing (*r *=* *0.87, *p *<* *0.001), and drought (*r *=* *0.16, *p *=* *0.098; Figure 4b).

## 4 DISCUSSION

### 4.1 Identify‐dependent responses of PFGs to grazing and drought

Plant functional group (PFG) identity is essential for understanding plant response to grazing intensities and climatic change (i.e., variations of rainfall both intra‐ and interannually). Based on our experiment, drought decreased the relative species abundance of annuals and biennials, but increased perennial bunchgrasses, perennial rhizome grass, and perennial forbs, respectively (Figure 1). However, grazing tended to increase annuals and biennials while decreasing the abundance of perennial forbs in both the drought and nondrought year, which is consistent with previous studies (Díaz et al., 2007; Hadar, Noy‐Meir, & Perevolotsky, 1999; Lavorel et al., 1997; McIntyre & Lavorel, 2001; Zheng et al., 2015). These results may have occurred because annuals and biennials tend to be characterized by short and small individuals, low palatability and low nutrition (Milchunas et al., 1988; Noy‐Meir et al., 1989; Wang, Liu, Hao, & Liang, 1996; Yan et al., 2015), such as *Salsola collina* Pall. and *Chenopodium aristatum* L. (Supporting Information Table S1), whereas perennial forbs are more palatable or higher in nutrients (e.g., *Legumes*,* Liliaceae*, and *Compositae* plants; Wang, Wang, He, Liu, & Hodgkinson, 2010; Bai et al., 2012). Moreover, our study indicated that the effects of grazing intensities on perennial grasses (both bunch and rhizome grasses) were not linear in the nondrought year. This might have occurred because perennial grasses—as dominant PFGs in the typical steppe on the Mongolian Plateau (Li et al., 2015; Ma et al., 2010; Pan et al., 2016)—are resistant to grazing because of their acquisitive‐conservative competition for resources (Bai et al., 2012; Zheng et al., 2015). This is also likely because the dominant PFGs were more highly sensitive to changes in environmental variation than to grazing intensities (Milchunas et al., 1993). However, dominant PFGs were susceptible to grazing in a California grassland (Stein et al., 2016); a further explanation for this argument is the viewpoint that the classification of plant functional type should be based on specific regions with different climatic and grazing historical scenarios (Díaz et al., 2007; Lavorel et al., 1997).

Grazing intensity and drought also altered the relationships among the PFGs. Specifically, our results suggested that annuals and biennials were negatively correlated with other PFGs (Figure 3). Livestock generally favored perennial grasses or forbs (McNaughton, 1985; Milchunas et al., 1988; Wang et al., 2010). Meanwhile, the less‐ or nonfavored annuals and biennials reproduce and develop by competing with others for the relatively “superfluous” resources (Wang et al., 1996), which are space and sufficient light and water resources unused by the shrinking perennial plants (Jameson, 1963), as well as extra nutrients from herbivore’ excretions (Turner, Seastedt, & Dyer, 1993). Moreover, annuals and biennials have higher rates of germination than perennial plants (Freas & Kemp, 1983; Mulroy & Rundel, 1977), and can quickly outcompete and colonize this system (Wang et al., 1996). However, the perennial grasses tend to be competitively dominant to other PFGs (Bai et al., 2012; Zheng et al., 2015), represented by a negative correlation with perennial forbs in our study. Finally, plants can have a positive interaction (e.g., facilitation) with other plants in conditions of high stress (Maestre et al., 2009), which could be a reason why perennial bunchgrasses were positively correlated with perennial rhizome grass in our study.

### 4.2 Regulating the CS and BEF by the PFGs

Herbivore grazing alters community composition and structure and changes ecosystem functions not only via the mechanisms of direct feeding, ingesting, and trampling (Eldridge et al., 2016; McNaughton, 1979; Milchunas et al., 1988), but also by indirectly regulating plant functional groups (Hadar et al., 1999; McIntyre & Lavorel, 2001; Papanikolaou et al., 2011; Pérez‐Camacho et al., 2012; Stein et al., 2016). First, our results suggested that all PFGs represented negative correlations with plant species richness (Figure 4a). Because species richness was tallied as the total number of species in the whole of PFGs, a change in any one of the relative species abundance of PFGs would have primarily altered the comparison of the other PFGs in the plant community. Additionally, these contributions of PFGs on species richness appeared to be associated with climatic conditions (Li et al., 2015; Pan et al., 2016; Yan et al., 2015), and were highly identity‐dependent in grazed grasslands (Díaz et al., 2007; Lavorel et al., 1997; Noy‐Meir, 1995; Su et al., 2017). Another possible reason behind the negative correlation may be that plant species or PFGs have strong interactions (competition or facilitation) under grazing or drought conditions (Maestre et al., 2009; Milchunas et al., 1988), suggesting that interactions with PFGs could play a major role in maintaining plant species richness.

Grazing and drought affected plant stand density directly and indirectly, while the correlation between drought and plant stand density seemed in insignificant. This supports our expectation that the effect of drought on plant stand density is indirect via regulating the interactions of PFGs rather than direct via changing it (Figure 4). This may have resulted from an increase in the abundance of annuals and biennials, which may have compensated for density loss. Alternatively, grazing and drought distinctly affected plant canopy height. Grazing can damage the canopy directly (McNaughton, 1979; Milchunas et al., 1988; Noy‐Meir, 1995; Su et al., 2017). However, the drought substantially reduced the annual and biennial plants, which are sensitive to the variation in rainfall (Yan et al., 2015), and consequently caused an increase in perennial plants, which are taller and bigger (Li et al., 2015; Ma et al., 2010; Pan et al., 2016), or more stress‐tolerant (Bai et al., 2012; Zheng et al., 2015; i.e., indirectly increased height).

Finally, the effect of grazing and drought on ecosystem functions was regulated by the interactions of PFGs. Numerous previous studies have suggested that grazing influence on species diversity and functional diversity were associated with environmental conditions (Collins et al., 1998; Hallett, Stein, & Suding, 2017; Noy‐Meir, 1995; Olff & Ritchie, 1998; Pérez‐Camacho et al., 2012). However, these studies did not report the effects of grazing and drought on functional diversity and species diversity in the context of regulating PFGs. We found that grazing decreased perennial forbs but not perennial bunchgrasses. Drought increased perennial forbs and perennial bunchgrasses, while the functional diversity was negatively associated with perennial bunchgrasses and perennial forbs. Additionally, species diversity was negatively correlated with perennial rhizome grass, which is one of the dominant species (*Leymus chinensis* (Trin.) Tzvel); the aboveground biomass was positively associated with species diversity and negatively correlated with functional diversity. These findings are all in agreement with previous studies (McNaughton, 1985; Wu, Wurst, & Zhang, 2016; Zhu, Jiang, & Zhang, 2016) where grazing was reported to play a secondary role in plant primary production (Biondini et al., 1998). However, one study found that drought effects were similar to or less than grazing, and it was not the main driver of grassland productivity (Koerner & Collins, 2014). Here we found that grazing was the primary factor leading to the decrease in primary production compared with rainfall variations, and that drought appeared to escalate these negative effects. Despite increased annuals and biennials mitigating the aboveground biomass loss through grazing, the grasslands remained vulnerable to grazing under the drought condition (Dangal et al., 2016; Eldridge et al., 2016).

In sum, we found that the responses of PFGs to herbivore grazing and drought were identity‐dependent. Herbivores distinctly favored perennial forbs, while drought reduced the relative species abundance of annuals and biennials and raised the abundance of perennial bunchgrasses and perennial rhizome grass. These reasons may help explain our major finding—that grazing and drought shaped or changed ecosystem structure and functioning via regulation of plant functional group composition change. Moreover, grazing consistently mediated community structural architecture (i.e., plant stand density and canopy height) while drought reduced community composition physiognomy (i.e., plant species richness). The grazing effects on species diversity, functional diversity and aboveground biomass appeared nonlinear; drought escalated grazing effects on primary production. Further comprehensive mechanisms on species or plant functional groups’ interactions in maintaining ecosystem stability in grazed grasslands under different climatic conditions should be prioritized in future research.

## CONFLICT OF INTEREST

None declared.

## AUTHORS’ CONTRIBUTIONS

ML, ZL, and CL conceived and designed the experiments. ML and XW conducted the field data collection. ML, JC, ESG, XB, ZL, and CL analyzed the data and developed the manuscript.

## DATA ACCESSIBILITY

All data will be uploaded in a repository once accepted.
