# Supplementary material for: Grazing effect on grasslands escalated by abnormal precipitations in Inner Mongolia
Source: Ecol Evol. 2018 Jul 22;8(16):8187–96. doi: 10.1002/ece3.4331 (PMC6144992; doi:10.1002/ece3.4331)
Supplement: Supplementary file 1 [file ECE3-8-8187-s001.docx]

**Table S1.** Species (total=65) and their functional groups (PFG) recorded in our sampling plots in a typical steppe on the Mongolian Plateau. PB - perennial bunchgrasses; PR- perennial rhizome grass, PF - perennial forbs; AB - annuals and biennials; SS - shrubs and semi-shrubs.

| No. | Family | Genera | Name | PFG |
| --- | --- | --- | --- | --- |
| 01 | *Amaranthaceae* | *Amaranthus* | *A. retroflexus* L. | AB |
| 02 | *Boraginaceae* | *Lappula* | *L. myosotis* V. Wolf | AB |
| 03 | *Chenopodiaceae* | *Axyris* | *A. amaranthoides* L. | AB |
|  |  | *Chenopodium* | *C. acuminatum* Willd. | AB |
|  |  |  | *C. aristatum* L. | AB |
|  |  |  | *C. glaucum* L. | AB |
|  |  | *Corispermum* | *C. declinatum* Steph. ex Stev. | AB |
|  |  | *Salsola* | *S. collina* Pall. | AB |
| 04 | *Compositae* | *Artemisia* | *A. frigida* Willd. | SH |
|  |  |  | *A. pubescens* | PF |
|  |  |  | *A. sieversiana* Ehrhart ex Willd. | AB |
|  |  |  | *A. sphaerocephala* Krasch. | SH |
|  |  | *Heteropappus* | *H. altaicus* (Willd.) Novopokr. | PF |
|  |  | *Saussurea* | *S. japonica* (Thunb.) DC. | AB |
|  |  | *Serratula* | *S. centauroides* L. | PF |
| 05 | *Cruciferae* | *Dontostemon* | *D. micranthus* C. A. Mey. | AB |
|  |  | *Lepidium* | *L. apetalum* | AB |
|  |  | *Thlaspi* | *T. thlaspidioides* (Pall.) Kitag. | PF |
| 06 | *Cyperaceae* | *Carex* | *C. korshinskyi* Kom. | PF |
| 07 | *Euphorbiaceae* | *Euphorbia* | *E. esula* L. | PF |
|  |  |  | *E. humifusa* Willd. ex Schlecht. | PF |
| 08 | *Fabaceae* | *Achnatherum* | *A. sibiricum* (L.) Keng | PB |
|  |  | *Agropyron* | *A. cristatum* (L.) Gaertn. | PB |
|  |  | *Chloris* | *C. virgata* Sw. | AB |
|  |  | *Cleistogenes* | *C. squarrosa* (Trin.) Keng | PB |
|  |  | *Eragrostis* | *E. pilosa* (L.) Beauv. | AB |
|  |  | *Leymus* | *L. chinensis* (Trin.) Tzvel. | PR |
|  |  | *Koeleria* | *K. cristata* (L.) Pers. | PB |
|  |  | *Poa* | *P. annua* L. | PB |
|  |  | *Setaria* | *S. viridis* (L.) Beauv. | AB |
|  |  | *Stipa* | *S. grandis* P. Smirn. | PB |
|  |  |  | *S. krylovii* Roshev. | PB |
| 09 | *Geraniaceae* | *Erodium* | *E. stephanianum* Willd. | PF |
| 10 | *Iridaceae* | *Iris* | *I. tenuifolia* | PF |
| 11 | *Labiatae* | *Dracocephalum* | *D. moldavica* L. | AB |
|  |  | *Phlomis* | *P. umbrosa* | PF |
|  |  | *Scutellaria* | *S. baicalensis* Georgi | PF |
| 12 | *Liliaceae* | *Allium* | *A. anisopodium* Ledeb. Var. anisopodium | PF |
|  |  |  | *A. bidentatum* | PF |
|  |  |  | *A. chrysanthum* Regel | PF |
|  |  |  | *A. mongolicum* Regel | PF |
|  |  |  | *A. polyrhizum* Turcz. ex Regel | PF |
|  |  |  | *A. ramosum* L. | PF |
|  |  |  | *A. tenuissimum* L. | PF |
|  |  | *Anemarrhena* | *A. asphodeloides* Bunge | PF |
| 13 | *Linaceae* | *Linum* | *L. stelleroides* Planch. | AB |
| 14 | *Plantaginaceae* | *Plantago* | P. asiatica L. | AB |
| 15 | *Plumbaginaceae* | *Limonium* | *L. bicolor* (Bag.) Kuntze | PF |
| 16 | *Poaceae* | *Astragalus* | *A. galactites* Pall. | PF |
|  |  |  | *A. melilotoides* Pall. | PF |
|  |  | *Caragana* | *C. microphylla* Lam. | SH |
|  |  | *Gueldenstaedtia* | *G. verna* (Georgi) Boriss. subsp. *multiflora* (Bunge) Tsui | PF |
|  |  | *Melissitus* | *M. ruthenica* | PF |
|  |  | *Oxytropis* | *O. myriophylla* (Pall.) DC. | PF |
|  |  | *Thermopsis* | *T. lanceolata*R. Br. | PF |
| 17 | *Polygonaceae* | *Polygonum* | *P. aviculare* L. | AB |
| 18 | *Primulaceae* | *Androsace* | *A. umbellata* (Lour.) Merr. | AB |
| 19 | *Ranunculaceae* | *Thalictrum* | *T. petaloideum* L. | PF |
|  |  |  | *T. squarrosum* Steph. | PF |
| 20 | *Rosaceae* | *Potentilla* | *P. bifurca* L | PF |
|  |  |  | *P. tanacetifolia* Willd. ex Schlecht. | PF |
|  |  | *Sibbaldia* | *S. procumbens* L. | PF |
| 21 | *Rutaceae* | *Haplophyllum* | *H. dauricum* (L.) G. Don | PF |
| 22 | *Scrophulariaceae* | *Cymbaria* | *C. dahurica* L. | PF |
| 23 | *Umbelliferae* | *Saposhnikovia* | *S. divaricata* (Trucz.) Schischk. | PF |
